# A review of the costs of delivering maternal immunisation during pregnancy

**DOI:** 10.1016/j.vaccine.2020.07.050

**Published:** 2020-09-11

**Authors:** Simon R Procter, Omar Salman, Clint Pecenka, Bronner P Gonçalves, Proma Paul, Raymond Hutubessy, Philipp Lambach, Joy E Lawn, Mark Jit

**Affiliations:** a Department of Infectious Disease Epidemiology, London School of Hygiene and Tropical Medicine, London, United Kingdom; b Center for Vaccine Innovation and Access, PATH, Seattle, Washington, United States; c Department of Immunization, Vaccines and Biologicals, World Health Organization, Geneva, Switzerland; d Modelling and Economics Unit, Public Health England, London, United Kingdom; e School of Public Health, University of Hong Kong, Hong Kong SAR, China

**Keywords:** Maternal, Pregnancy, Immunisation, Vaccination, Costs, Economics

## Abstract

**Background:**

Routine maternal immunisation against influenza and pertussis are recommended by the WHO to protect mother and child, and new vaccines are under development. Introducing maternal vaccines into national programmes requires an understanding of vaccine delivery costs – particularly in low resource settings.

**Methods:**

We searched Medline, Embase, Econlit, and Global Health for studies reporting costs of delivering vaccination during pregnancy but excluded studies that did not separate the vaccine purchase price. Extracted costs were inflated and converted to 2018 US dollars.

**Results:**

Sixteen studies were included, of which two used primary data to estimate vaccine delivery costs. Costs per dose ranged from $0.55 to $0.64 in low-income countries, from $1.25 to $6.55 for middle-income countries, and from $5.76 to $39.87 in high-income countries.

**Conclusions:**

More research is needed on the costs of delivering maternal immunisation during pregnancy, and of integrating vaccine delivery into existing programmes of antenatal care especially in low and middle-income countries.

## 1 Introduction

In the last decade maternal immunisation has become a rapidly expanding area of research and clinical practice to protect pregnant women and children from vaccine preventable diseases [1]. In particular this has been motivated by the high mortality amongst neonates and young infants, with newborns accounting for almost half of all deaths in children under 5 years [2], and ending preventable newborn deaths by 2030 is a priority of the United Nations Sustainable Development Goals. Part of this disparity in outcomes is due to the burden of infectious diseases and higher risk posed by some infections during the first weeks or months of life before the commencement of routine childhood immunisation schedules. Vaccination of pregnant women offers a means to protect newborns by bridging this gap in immunity through the transfer of maternal antibodies *in utero* [3].

Maternal immunisation with Tetanus Toxoid (TT) was first introduced into the World Health Organisation (WHO) Extended Programme on Immunisation (EPI) in the 1970s and has been a key tool in subsequent efforts to eliminate the burden of maternal and neonatal tetanus. During the 2009 influenza H1N1 pandemic it was recognised that pregnant women experienced a higher risk of severe complications and death, which led the WHO’s Strategic Advisory Group of Experts (SAGE) on immunisation to identify pregnant women as the highest priority group for influenza vaccination [3], [4]. Around the same time a randomized trial conducted in Bangladesh showed that vaccinating pregnant women reduced the risk of influenza in their newborn infants [5]. In 2012, both the United States and United Kingdom (UK) adopted routine administration of Tetanus, Diphtheria and Acellular Pertussis (Tdap) vaccine during pregnancy to prevent pertussis in young infants [3]. Subsequently, the routine vaccination of pregnant women against pertussis and influenza are increasingly being recommended as part of national immunisation programmes [6].

There is growing interest in the potential for maternal immunisation using other vaccines such as pneumococcal conjugate (PCV), Haemophilus influenzae type B (HiB) and meningococcal vaccines, as well as the potential for new vaccines against Cytomegalovirus, Zika virus and malaria [7], [8]. Of particular note are vaccines being developed against group B streptococcus (GBS) and respiratory syncytial virus (RSV) [8], [9], which could become the first vaccines specifically designed and licensed for use in pregnancy to protect the unborn child. As these and other vaccines progress towards market an understanding of the delivery costs in different settings will be important for assessing the cost-effectiveness and financial impact of their introduction. Furthermore, these costs may differ from vaccine delivery in other populations such as routine childhood immunisation, for example there may be costs associated with setting up new delivery channels outside of existing EPI programmes and costs of integrating delivery within antenatal care (ANC) programmes.

The objective of this study was to review the literature on the financial and economic non-vaccine costs of delivering maternal immunisation and incorporating it into routine, nation-wide vaccination programmes. The findings from this review can be used to inform economic evaluations of maternal immunisation, including current vaccines such as influenza and PCV, and prospective vaccines against RSV and GBS.

## 2 Methods

We performed a systematic search of the global literature to identify studies that reported the costs of delivering maternal immunisations during pregnancy. Searches were performed across Medline, Embase, Econlit, and Global Health bibliographic databases. The search strategy incorporated a mixture of index terms and key words which combined the concepts of: (i) vaccination; (ii) pregnancy; (iii) costs, and (iv) diseases with existing or pipeline vaccines recommended for maternal immunisation. The list of included vaccines was based on a recent review by Omer [3], and the full search strategy and complete list of search terms are provided in the supplementary material.

We included any English language articles that reported estimates of the non-vaccine cost of vaccinating pregnant women and excluded studies that reported only a vaccine purchase price, or for which the vaccine delivery and purchase costs could not be separated. We excluded articles where cost estimates were not specific to our population of interest, including estimates for vaccinating women of childbearing age that did not distinguish women who were pregnant. Studies were included regardless of the data source for cost estimation including estimates based on primary and secondary data, government tariffs, expert opinion, and assumption.

Search results were combined using reference management software (Mendeley) and any duplicate records were removed. Two reviewers (OS and SP) screened articles using titles and abstracts, followed by full text screening of potentially eligible studies to determine the final list of included articles. We recorded descriptive study characteristics including the country, currency and year of cost estimates, type of vaccine, and details of source data and methodology for cost estimation. We extracted reported non-vaccine delivery costs related to immunisation of pregnant women – this included total programme costs, cost per dose, and any component costs which were separately reported. In addition we also recorded the reported price of purchasing a vaccine dose.

Extracted costs were inflated to 2018 currency values using national GDP deflators [10] from the World Bank, and figures reported in local currency units were then converted into United States Dollars (USD) using the average 2018 exchange rates [11]. Results were also converted to international dollars (I$) using Purchasing Price Parity (PPP) conversion rates [12]. Where possible we calculated cost per dose if this was not already reported by the study authors.

## 3 Results

**Fig. 1:**
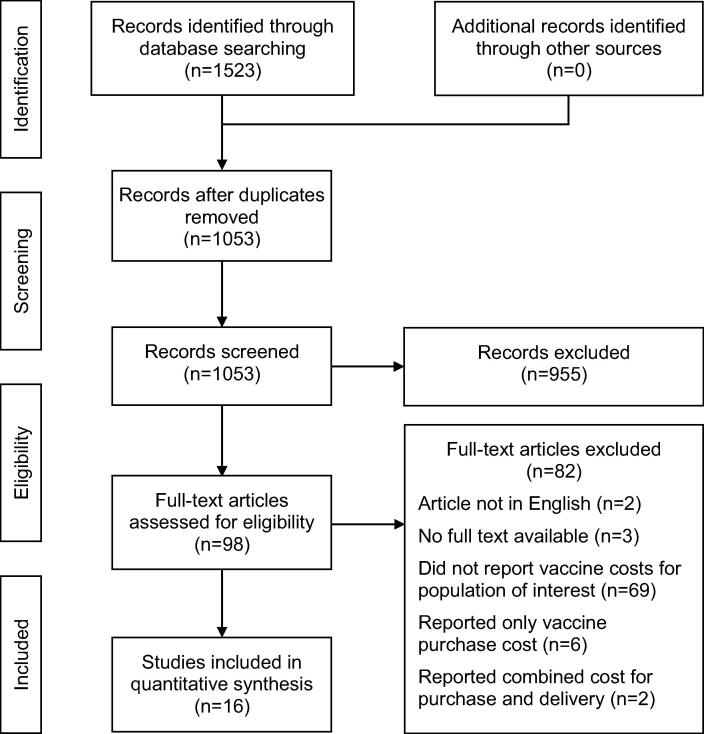
Flow diagram showing the screening process used to identify included studies.

Our initial search resulted in 1523 records across the different databases, and 1053 unique articles once duplicates were removed. Following the first stage of screening we identified 98 potentially eligible articles, and after full-text screening there were 16 articles that met the inclusion criteria. The flow diagram summarising the results of our search and screening is shown in Fig. 1.

Key characteristics of the articles included are summarised in Table 1, and further details are provided in the supplementary material. Eleven studies were from high-income countries (HICs) [13], [14], [15], [16], [17], [18], [19], [20], [21], [22], [23], with three studies in middle-income countries (MICs) [13], [24], [25] and only two studies in low-income countries (LICs) [26], [27]. The studies covered four existing vaccines – Tetanus Toxoid, Tdap, Influenza, and Hepatitis E – as well as the hypothetical introduction of a new vaccine against GBS. Six studies reported costs for delivering vaccines as part of routine ANC visits, while the remaining studies did not make this explicit. Although we applied no date limits to our search, all but one of the articles were published since 2010 reflecting the recent and growing interest in the introduction of maternal vaccines to national programmes.

**Table 1:** Descriptive characteristics of included studies.

Lead author	Year	Country	Vaccine	Study type	Source of cost estimation	Primary data on resource utilisation	Ingredients based approach	Vaccine delivery during routine ANC visit?
Atkins	2016	USA	Tdap	EE	Literature			Not specified
Baguelin	2010	UK	Influenza	EE	Government tariff			Not specified
Berman	1991	Indonesia	TT	EE	Primary data	X	X	Yes
Fernández-Cano	2015	Spain	Tdap	EE	Government tariff			Not specified
Garcia	2016	Spain	Influenza	EE	Government tariff			Not specified
Giorgakoudi	2018	UK	GBS	EE	Government tariff			Not specified
Jit	2010	UK	Influenza	EE	Government tariff + secondary data			Not specified
Kim	2014	South Africa	GBS	EE	Secondary data + expert opinion		X	Yes
Kim	2017	USA	GBS	EE	Secondary data + expert opinion		X	Yes
Pecenka	2017	Malawi	Influenza	Cost study	Primary + secondary data	X	X	Yes
Sartori	2016	Brazil	Tdap	EE	Secondary data		X	Yes
Skedgel	2011	Canada	Influenza	EE	Government tariff / unpublished estimate^a^			Not specified
Terranella	2013	USA	Tdap	EE	Assumption			Not specified
van Hoek	2016	UK	Tdap	EE	Assumption			Not specified
Xu	2016	USA	Influenza	EE	Literature			Yes
Zhao	2016	China	Hepatitis E	EE	Literature			Not specified

EE = Economic Evaluation; TT = Tetanus Toxoid; Tdap = Tetanus, diphtheria & acellular pertussis

a Private communication from Nova Scotia Department of Health Promotion and Protection.

Globally the estimated cost per dose of delivering maternal immunisation in 2018 USD ranged from $0.55 in Malawi [26] to $39.87 in Canada [28]. Reported costs ranged from $0.55 to $0.64 for LICs, $1.25 to $6.55 for MICs, and $5.76 to $39.87 in HICs. As variation in costs across income levels will result from differences in the purchasing power of each dollar, we also present results in Table 2 using I$ to facilitate comparisons between countries. All of the studies reported costs from the health care (payer / provider) perspective; however, this perspective included different costs in different studies.

**Table 2:** Reported cost per dose for delivery of maternal vaccination.

Study Author	Year	Country	Delivery cost per dose (range)				Vaccine price
			2018 USD		2018 I$		2018 USD
**Low-income**							
Pecenka	2017	Malawi	0.55	(0.55 – 0.83)	1.81	(1.81 – 2.73)	3.06
Berman^a^	1991	Indonesia	0.64	(0.44 – 2.80)	2.14	(1.48 – 9.40)	0.28

**Upper-middle income**							
Zhao	2016	China	1.25		2.33		19.77
Sartori	2016	Brazil	2.14		3.86		13.96
Kim^b^	2014	South Africa	6.55	(3.28 – 9.83)	14.06	(7.04 – 21.10)	23.01

**High-income**							
Skedgel^c^	2011	Canada	5.76		5.99		3.23
Baguelin	2010	UK	8.30		8.89		16.92
van Hoek	2016	UK	10.39		11.12		18.54
Fernández-Cano	2015	Spain	10.46		13.68		12.16
Jit	2010	UK	11.87	(8.69 – 16.33)	12.71	(9.31 – 17.48)	10.22
Garcia^d^	2016	Spain	12.85		16.81		14.51
Giorgakoudi	2018	UK	13.87		14.84		81.79
Xu	2016	USA	17.15	(8.79 – 17.15)	17.15	(8.79 – 17.15)	13.41
Terranella	2013	USA	22.54	(11.27 – 33.81)	22.54	(11.27 – 33.81)	42.37
Atkins	2016	USA	24.20		24.20		23.15
Kim^e^	2017	USA	25.96	(12.13 – 40.22)	25.96	(12.13 – 40.22)	116.41
Skedgel^f^	2011	Canada	39.87		41.51		3.23

a We extracted the delivery cost per pregnant woman receiving two doses of TT from figure 3 of Berman et al. for each sub-district and converted to cost per dose by dividing by 2; to calculate the average cost across all sub-districts we used total number of TT doses per district reported in Table 1 of Berman et al. (in total 2293 women received two doses).

b Vaccine price based on mid-point of assumed public sector price range

c Delivery cost based on unpublished estimate of average cost of vaccine delivery in a public health clinic.

d Vaccine price for quadrivalent influenza vaccine.

e Vaccine price based on assumed public sector price.

f Delivery cost based on government fee schedule for delivery by family physician.

Only two studies undertook detailed costing of maternal vaccine programmes through the collection of primary data on resource use and costs. Berman and co-workers estimated the financial cost to the government of $0.64 per dose for an on-going tetanus maternal immunisation programme provided as part of routine antenatal care (ANC) in Aceh province, Indonesia [27]. More recently, Pecenka et al. estimated the financial cost to be $0.55 per dose for incorporating a prospective programme of seasonal influenza immunisation alongside existing antenatal care provision in Malawi [26].

The majority of the articles described economic evaluations where detailed estimation of vaccination delivery costs was not a primary research objective and differed in their approaches to cost estimation. In addition to the studies by Berman and Pecenka, only three other studies by Kim [13], [23] and Sartori [25] used an ingredients-based approach in which some disaggregation of resource use was used to estimate an overall vaccine delivery cost (a summary of individually reported cost components is provided in the supplementary material) [13], [23], [25], [26], [27]. Six studies in the UK, Spain and Canada estimated costs using government tariffs or fees for a physician visit [14], [28], [16], [17], [18], [19]. The remaining five articles either reported costs based on the authors’ assumption or cited estimates of vaccine administration costs from the literature, however the sources of these estimates were not specific to vaccinating pregnant women [15], [24], [20], [21], [22].

## 4 Discussion

Methodological differences between studies may have lead to important differences in estimated costs. For example, where estimates were based on tariffs or fees these service payments might reflect the marginal cost from the perspective of the healthcare payer. However, they may not necessarily represent the true economic cost of vaccine delivery, which will depend on the actual resources used in the providing the service. As an illustration, Jit et al. estimated the cost of delivering maternal influenza vaccine in the UK to be $11.87 per dose based on a National Health Service (NHS) tariff payment [17]. In contrast estimates based on the appointment duration and valuation of nurses’ time ranged from $8.69 to $16.33 per dose depending on the grade of nurse delivering the vaccine.

A further consideration is that estimates based only on tariffs and fees are likely to neglect any costs of introducing a new vaccine programme that are borne centrally by the government or health service. Two of the studies that used an ingredients-based approach reported costs of programme introduction including expenditure on planning, training, social mobilisation and capital investment in cold chain capacity [13], [26]. In the South African study by Kim et al. these costs accounted for $1.44 (22%) of the delivery costs per dose based on estimates using previously published analysis from other vaccine programmes. Figures reported by Pecenka et al. showed that introduction costs were $0.36 (66%) of the total non-vaccine costs per dose, and that the start-up costs were ~ 3 times higher in the first year of the programme. These findings highlight the importance of these costs and of considering whether or not they have been included when drawing comparisons across studies.

The relative contribution of fixed costs – which do not change in the short-run – and variable costs that increase with the number of vaccinations can be an important determinant of programme efficiency and the overall cost per dose. For example, in their base case analysis, Pecenka et al. found the financial cost per dose was $0.55, but due to the effect of fixed programme costs this increased to $0.83 per dose in a sensitivity analysis which assumed lower vaccine coverage. Similarly, Berman et al. reported an average vaccine delivery cost of $0.64 per dose, but this ranged across sub-districts from $0.44 to $2.80 per dose. The authors found that this variation was primarily driven by the amortisation of fixed programme costs across substantially different numbers of women vaccinated in each sub-district. These fixed costs did not depend on how many women were vaccinated and included both capital investment in equipment as well as recurrent expenditure on wages and allowances for certain personnel.

For the five studies in our sample conducted in LMICs our findings for maternal immunization are broadly similar to the results of a recent review on the costs of immunisation delivery in LMICs more generally, which reported costs per dose ranging from $0.16 to $2.54 (in 2016 USD) [29]. Only Kim’s estimate for South Africa fell outside this range [13]. However, given the small number of studies we identified it is difficult to draw general conclusions about the extent to which estimates from other types of vaccine programme can inform the cost of maternal immunisation. More primary data are needed to establish how these costs vary across different income settings, and across different health systems with different levels of existing antenatal care provision. There is also a need for more evidence on whether general tariffs and fees are a reasonable proxy of vaccine delivery costs amongst pregnant women. Good cost estimates are important for informing decisions on the cost-effectiveness of adopting existing and new maternal vaccines.

For decision makers the relative importance of non-vaccine delivery costs may be influenced by the vaccine purchase price. Amongst our included studies we found non-vaccine costs as a proportion of the total cost ranged from 15% to 70% in LICs, 6% to 22% in MICs, and 14% to 93% in HICs. The broad ranges largely reflect the variation in vaccine purchase price (Table 1), which in general are higher for newer vaccines. Although high purchase prices for new vaccines may be cost-prohibitive in lower-income settings, the non-vaccine delivery costs could still be an important determinant in influencing expansion of maternal immunisation where purchase costs are funded or subsidised through external donor support, or for vaccines that are relatively cheap such as Tdap or TT vaccines.

Of the studies we found only one used a dedicated costing tool [30], although several tools exist to support costing of immunisation programmes such as the WHO FLUtool, the comprehensive Multi-Year Planning (cMYP) costing tool, and the ONE Health tool [30], [31], [32]. To improve the comparability of cost estimates future research should follow immunisation costing guidelines and more standardisation is needed in terms of methodological approach and transparency in the reporting of findings.

Another limitation in the literature was the lack of studies examining costs from the perspective of pregnant women and their families. The costs of transport and time spent attending appointments may act as a barrier to the uptake of maternal immunisation, but these additional costs may be avoided where maternal vaccines can be delivered as part of existing ANC visits. In this case the health benefits of introducing new maternal vaccines could even act as an incentive helping to boost coverage of wider antenatal care packages.

Our study is subject to several limitations. First, we have not formally appraised the quality of the included studies for factors which may bias the reported cost estimates. However, we do report study methodology - there is a generally accepted hierarchy in which estimates using ingredients-based costing are usually considered better than those based on tariffs [33], and the quality of estimates based purely on assumption cannot be judged. Second, we included only studies that were written in English and excluded articles in other languages. Finally, we searched only the peer-reviewed academic literature, and therefore do not include information that may exist in the grey literature.

## 5 Conclusion

We found few studies in the academic literature that reported estimates of the costs of delivering maternal immunisation to pregnant women, and only two studies that collected primary data on resource use. This highlights the need for further research to identify the economic and financial costs of delivering vaccinations during pregnancy. More detailed costing studies are needed that use primary data and micro-costing to establish whether the non-vaccine costs of maternal immunisation delivery differ from other immunisations programmes, and the extent to which such differences may vary by setting. Studies should consider the start-up costs of introducing new maternal immunisation programmes, and the costs of integrating delivery within existing antenatal care services. A good understanding of these costs is critical to informing the cost-effectiveness and financial impact of introducing new maternal vaccines, particularly in low-resource settings.

## Funding

10.13039/100000865The Bill & Melinda Gates Foundation, Seattle, WA (OPP1180644).

## Authorship

MJ, JEL, OS and SRP conceptualised the study. SRP, OS, MJ, and CP developed the methodology. OS and SRP conducted the review, analysed the results and drafted the original manuscript. All authors critically reviewed the manuscript and approved the final version.

## Disclaimer

Hutubessy R and Lambach P work for the World Health Organization. The authors alone are responsible for the views expressed in this publication and they do not necessarily represent the decisions, policy or views of the WHO.

## Declaration of Competing Interest

The authors declare that they have no known competing financial interests or personal relationships that could have appeared to influence the work reported in this paper.
