# Supplementary material for: A review of the costs of delivering maternal immunisation during pregnancy
Source: Vaccine. 2020 Sep 11;38(40):6199–204. doi: 10.1016/j.vaccine.2020.07.050 (PMC7482437; doi:10.1016/j.vaccine.2020.07.050)
Supplement: Supplementary Data 1 [file mmc1.docx]

**Supplementary material**

**A review of the costs of delivering maternal immunisation during pregnancy**

**Appendix 1: Search strategy**

Four electronic bibliographic databases were searched: Embase, Medline, Global Health and Econlit. The search strategy combined four concepts to identify studies that measure the costs of implementing vaccinations: (i) vaccination, (ii) pregnancy, (iii) costs, and (iv) vaccine-preventable diseases through maternal immunisation.

The following search strategy was used our selected databases:

1. vaccination/ or mass vaccination/

2. vaccin* or immunis* or immuniz*

3. mother or matern* or pregnan* or antepartum or antenatal or puerperal or peripartum or intrapartum

4. "Costs and Cost Analysis"/ or Economics, Hospital/

5. cost* or expenditure or economic* or CEA or CUA or CBA

6. group B streptococcus or respiratory syncytial virus or GBS or RSV

7. Tetanus Toxoid/ or Diphtheria-Tetanus Vaccine/ or Diphtheria-Tetanus-Pertussis Vaccine/ or Diphtheria-Tetanus-acellular Pertussis Vaccines/

8. influenza* or tetanus or diphtheria or pertussis or IIV* or Tdap or TT* or TD or TIV or TTV or DTP or DTaP or DTwP

9. 1 or 2

10. 4 or 5

11. 6 or 7 or 8

12. 3 and 9 and 10 and 11

**Appendix 2: Extracted data for included studies**

| **Lead author** | **Year published** | **Country** | **Vaccine type** | **Study type** | **Description** | **Primary data on resource use** | **Reporting currency (ISO code)** | **Currency year** | **Costs per dose in 2018 USD**  **(range)** | **Total programme costs in 2018 USD** | **Ref.** |
| --- | --- | --- | --- | --- | --- | --- | --- | --- | --- | --- | --- |
| Atkins | 2016 | USA | Tdap | EE | This study used a dynamic transmission model to estimate the cost-effectiveness of vaccinating pregnant women against pertussis compared to post-partum parental vaccination from a provider perspective. The cost for vaccine administration was based on a hospital study of a pertussis cocooning strategy by Healy et al. (1) |  | USD | 2013 | 24.20  This was based on a literature value by Healy that reported the cost per dose to vaccinate post-partum women including “the cost of overhead, faculty, and nursing personnel required to administer the program.” (1) No cost breakdown was provided. |  | (2) |
| Baguelin | 2010 | UK | Influenza | EE | This study used a dynamic transmission model to estimate the cost-effectiveness of different vaccination strategies against pandemic influenza in high risk populations including pregnant women. The analysis was from the health care provider perspective, and vaccine delivery costs were based on previous published GP tariff payments for swine flu. |  | GBP | 2008 | 8.30 |  | (3) |
| Berman | 1991 | Indonesia | TT | EE | This study compared the cost-effectiveness of maternal TT vaccination during routine antenatal care vs a mass campaign. Primary data on resource use and costs was collected retrospectively from a routine on-going programme. Costs of vaccine administration were estimated from the health system perspective. | X | IDR | 1985/ 1986 | 0.64 (0.44 to 2.80) |  | (4) |
| Fernández-Cano | 2015 | Spain | Tdap | EE | This study estimated the benefit to cost ratio for pertussis vaccination during pregnancy and a cocooning vaccination strategy from the health system perspective. The cost of vaccine administration was based on “cost according to the 2012 Framework Agreement in Catalonia.” |  | EUR | 2015 | 10.46 |  | (5) |
| Garcia | 2016 | Spain | Influenza | EE | This modelled the cost-effectiveness of introducing quadrivalent influenza vaccine to high risk groups including pregnant women. The costs of vaccine administration were based on regional tariffs (payer perspective.) |  | EUR | 2014 | 12.85 |  | (6) |
| Giorgakoudi | 2018 | UK | GBS | EE | This study modelled the cost-effectiveness of introducing a hypothetical maternal vaccine against GBS from the healthcare provider perspective. The cost of vaccine administration was based on the NHS tariff payment for vaccination by GP practices. |  | GBP | 2015 | 13.87 |  | (7) |
| Jit | 2010 | UK | Influenza | EE | This study modelled the cost-effectiveness of vaccinating pregnant women against seasonal influenza from a health service perspective. Costs for vaccine administration were based on tariff payments to GPs for vaccine administration. According to the methods “This was varied between £5.50 (10 min of client contact time for a band 5 practice nurse) to £10.33 (10 min for a midwife, i.e. a band 7 advanced nurse).” Staff time was valued using unit costs from Curtis et al.(8) |  | GBP | 2008 | 16.33 (8.96 to 28.46) |  | (9) |
| Kim | 2014 | South Africa | GBS | EE | This study modelled the cost-effectiveness of introducing a hypothetical maternal vaccine against GBS from the perspective of the South African health system. Vaccine programme costs were estimated using an ingredients-based approach with different cost items estimated based on expert opinion, literature and published data. |  | USD | 2010 | 6.55 (3.28 to 9.83)  **Composed of:**  Cold chain: 1.04  Program activities (social mobilization, training and program management,  surveillance and monitoring): 0.40  Costs for personnel (nurse) time and vehicles and transportation were also included but not reported separately. |  | (10) |
| Kim | 2017 | USA | GBS | EE | This study modelled the cost-effectiveness of introducing a hypothetical maternal vaccine against GBS from the perspective of the US health system. The costs of vaccine administration were estimated based on personnel time and vaccine supplies using expert opinion, literature and published data. |  | USD | 2013 | 25.96 (12.13 to 40.22)  **Composed of:**  Obs. & Gynae. counselling: 17.39  Nurse time: 5.90  Gloves: 0.05  Sharps disposal: 0.16  Alcohol wipe, band-aid, gauze: 0.33 |  | (11) |
| Pecenka | 2017 | Malawi | Influenza | Cost study | This study piloted a costing tool (the WHO Flu tool) for maternal influenza immunization programmes. Primary and secondary data were collected at the national, regional, district and facility levels on a prospective national immunization programme in Malawi. An ingredient-based approach was used to estimate the introduction and on-going financial and economic costs from the government perspective under different scenarios. | X | USD | 2015 | 0.55 (0.55 to 0.83) | Total over 5-years: 1,235,662  **Composed of:**  Microplanning: 168,640  Training: 159,120  Social mobilisation: 81,188  Supplementing cold chain: 423,201  Service delivery: 194,402  Vaccine supplies: 13,196  Information, education, and communication: 58,241  Monitoring & evaluation: 118,917  Other: 18,757 | (12) |
| Sartori | 2016 | Brazil | Tdap | EE | This study modelled the cost-effectiveness of introducing routine maternal pertussis immunization (Tdap) in Brazil. The cost of administering a vaccine dose was estimated using an ingredients-based approach using published cost data from a national survey. |  | USD | 2011 | 2.14  Composed of “transport and storage costs, personnel time and immunization-related supplies” but with no cost breakdown provided. |  | (13) |
| Skedgel | 2011 | Canada | Influenza | EE | This study compared the cost-effectiveness of target and universal maternal influenza strategies for pregnant women in Nova Scotia, Canada using a health system payer perspective. The study used two cost estimates for vaccine administration: cost of delivery by a family physician was based on the government fee schedule; delivery in a public health clinic was based on average vaccine delivery costs provided by the department of health promotion & protection. |  | CAD | 2010 | Delivered in public health clinic:  5.76  Delivered by family physician:  39.87  **Composed of:**  Physician fee: 25.29  Tray fee: 2.92  Injection fee: 11.67 |  | (14) |
| Terranella | 2013 | USA | Tdap | EE | This study used a cohort model to compare the cost-effectiveness of Tdap maternal immunisation vs a post-partum cocooning strategy to prevent infant pertussis. The cost of vaccine administration was based on the author’s assumption. |  | USD | 2011 | 22.54 (11.27 to 33.81) |  | (15) |
| van Hoek | 2016 | UK | Tdap | EE | This study modelled the cost-effectiveness of routine maternal vaccination against pertussis in England. The cost of vaccine administration as based on the author’s assumption. |  | GBP | 2016 | 10.39 |  | (16) |
| Xu | 2016 | USA | Influenza | EE | This study modelled the cost-effectiveness of seasonal influenza vaccination of pregnant women in the united states. The cost of vaccine administration in a private clinic was based on an article by Zhou et al. (17), which in turn cites a paediatric vaccine fee survey. |  | USD | 2013 | 17.15 (8.79 to 17.15) |  | ﻿(18) |
| Zhao | 2016 | China | Hepatitis E | EE | This study estimated the cost-effectiveness of hepatitis E vaccination of pregnant women in epidemic regions of China. The cost of vaccine delivery was based on an unpublished study by Xiu: "Disease Burden of Hepatitis E and Health Economic Evaluation of Vaccine Interventions in Dongtai Area" Southeatern university, 2011. |  | CNY | 2015 | 1.25 |  | (19) |
| EE = Economic Evaluation; TT = Tetanus Toxoid; Tdap = Tetanus, diphtheria & acellular pertussis; GBS = Group B Streptococcus; GBP = Pound Sterling; USD = United States Dollar; CNY = Yuan Renminbi; CAD = Canadian Dollar; EUR = Euro; IDR = Rupiah | | | | | | | | | | | |

**References**

1. Healy CM, Rench MA, Baker CJ. Implementation of Cocooning against Pertussis in a High-Risk Population. Clinical Infectious Diseases. 2011 Jan 15;52(2):157–62.

2. Atkins KE, Fitzpatrick MC, Galvani AP, Townsend JP. Cost-Effectiveness of Pertussis Vaccination During Pregnancy in the United States. Am J Epidemiol. 2016 Jun 15;183(12):1159–70.

3. Baguelin M, Hoek AJV, Jit M, Flasche S, White PJ, Edmunds WJ. Vaccination against pandemic influenza A/H1N1v in England: A real-time economic evaluation. Vaccine. 2010 Mar;28(12):2370–84.

4. Berman P, Quinley J, Yusuf B, Anwar S, Mustaini U, Azof A, et al. Maternal tetanus immunization in Aceh Province, Sumatra: the cost-effectiveness of alternative strategies. Soc Sci Med. 1991;33(2):185–92.

5. Fernández-Cano MI, Armadans Gil L, Campins Martí M. Cost–benefit of the introduction of new strategies for vaccination against pertussis in Spain: Cocooning and pregnant vaccination strategies. Vaccine. 2015 May;33(19):2213–20.

6. García A, Ortiz de Lejarazu R, Reina J, Callejo D, Cuervo J, Morano Larragueta R. Cost–effectiveness analysis of quadrivalent influenza vaccine in Spain. Human Vaccines & Immunotherapeutics. 2016 Sep;12(9):2269–77.

7. Giorgakoudi K, O’Sullivan C, Heath PT, Ladhani S, Lamagni T, Ramsay M, et al. Cost-effectiveness analysis of maternal immunisation against group B Streptococcus (GBS) disease: A modelling study. Vaccine. 2018 Nov;36(46):7033–42.

8. Curtis L, University of Kent at Canterbury, Personal Social Services Research Unit. Unit costs of health and social care 2009. Canterbury: Personal Social Services Research Unit; 2010.

9. Jit M, Cromer D, Baguelin M, Stowe J, Andrews N, Miller E. The cost-effectiveness of vaccinating pregnant women against seasonal influenza in England and Wales. Vaccine. 2010 Dec;29(1):115–22.

10. Kim S-Y, Russell LB, Park J, Verani JR, Madhi SA, Cutland CL, et al. Cost-effectiveness of a potential group B streptococcal vaccine program for pregnant women in South Africa. Vaccine. 2014 Apr;32(17):1954–63.

11. Kim S-Y, Nguyen C, Russell LB, Tomczyk S, Abdul-Hakeem F, Schrag SJ, et al. Cost-effectiveness of a potential group B streptococcal vaccine for pregnant women in the United States. Vaccine. 2017 Oct;35(45):6238–47.

12. Pecenka C, Munthali S, Chunga P, Levin A, Morgan W, Lambach P, et al. Maternal influenza immunization in Malawi: Piloting a maternal influenza immunization program costing tool by examining a prospective program. Turner SJ, editor. PLoS ONE. 2017 Dec 27;12(12):e0190006.

13. Sartori AMC, de Soárez PC, Fernandes EG, Gryninger LCF, Viscondi JYK, Novaes HMD. Cost-effectiveness analysis of universal maternal immunization with tetanus-diphtheria-acellular pertussis (Tdap) vaccine in Brazil. Vaccine. 2016 Mar;34(13):1531–9.

14. Skedgel C, Langley JM, MacDonald NE, Scott J, McNeil S. An incremental economic evaluation of targeted and universal influenza vaccination in pregnant women. Can J Public Health. 2011 Dec;102(6):445–50.

15. Terranella A, Asay GRB, Messonnier ML, Clark TA, Liang JL. Pregnancy Dose Tdap and Postpartum Cocooning to Prevent Infant Pertussis: A Decision Analysis. PEDIATRICS. 2013 Jun 1;131(6):e1748–56.

16. van Hoek AJ, Campbell H, Amirthalingam G, Andrews N, Miller E. Cost-effectiveness and programmatic benefits of maternal vaccination against pertussis in England. Journal of Infection. 2016 Jul;73(1):28–37.

17. Zhou F, Ortega‐Sanchez IR, Guris D, Shefer A, Lieu T, Seward JF. An Economic Analysis of the Universal Varicella Vaccination Program in the United States. J INFECT DIS. 2008 Mar;197(s2):S156–64.

18. Xu J, Zhou F, Reed C, Chaves SS, Messonnier M, Kim IK. Cost-effectiveness of seasonal inactivated influenza vaccination among pregnant women. Vaccine. 2016 Jun;34(27):3149–55.

19. Zhao Y, Zhang X, Zhu F, Jin H, Wang B. A preliminary cost-effectiveness analysis of hepatitis E vaccination among pregnant women in epidemic regions. Human Vaccines & Immunotherapeutics. 2016 Aug 2;12(8):2003–9.
